# Internal Jugular Vein Thrombosis: Unusual Diagnosis of a Cervical Mass

**DOI:** 10.7759/cureus.14516

**Published:** 2021-04-16

**Authors:** Rakan Saadoun, Susanne Reiser, Eva-Maria Risse, Ranim Bittar, Theresa Obermueller

**Affiliations:** 1 Department of Otorhinolaryngology, Head and Neck Surgery, University Hospital Marburg, Philipps-Universität Marburg, Marburg, DEU; 2 Department of Plastic Surgery, University of Pittsburgh, Pittsburgh, USA; 3 BG Trauma Center Ludwigshafen, Department of Hand, Plastic and Reconstructive Surgery, Burn Care Center, Hand and Plastic Surgery, Ruprecht Karls University Heidelberg, Ludwigshafen, DEU; 4 Faculty of Medicine, Damascus University, Damascus, SYR; 5 Department of Cardiology, Heart Center of Riverside, Riverside, USA; 6 Department of Otorhinolaryngology, Head and Neck Surgery, University Medical Centre Mannheim, Mannheim, DEU

**Keywords:** internal jugular vein thrombosis, neck mass, cerebral venous sinus thrombosis

## Abstract

Idiopathic internal jugular vein thrombosis (IJVT) is a rarity that we must quickly identify and manage, as it may have severe consequences such as cerebral venous sinus thrombosis (CVST). CVST might be fatal unless it is managed promptly. However, due to its rarity, clinicians are often unfamiliar with the presentation of this pathology.

We report an unusual finding of IJVT in a 53-year-old female patient who presented with a cervical mass on the left side to our otolaryngology outpatient clinic. A cervical ultrasound and computed tomography (CT) confirmed the diagnosis of IJVT. An extensive workout ruled out thrombophilia, CVST, cardiovascular diseases, head and neck cancers, and urinogenital tract neoplasms. The symptoms resolved under treatment with anticoagulation.

## Introduction

Idiopathic internal jugular vein thrombosis (IJVT) can be provoked or unprovoked. The former can be predisposed by ear-nose-throat (ENT) operations, head and neck neoplasms, and the placement of a central catheter. The unprovoked form is associated with paraneoplastic syndrome and ovarian hyperstimulation syndrome or it could be idiopathic IJVT [1-4]. Both forms represent a rarity, and there are no specific epidemiological studies about these entities [2-3].

## Case presentation

A 53-year-old patient presented with a cervical mass combined with a headache in our outpatient clinic. The swelling appeared three days before the presentation, and its size had been slightly increasing since then. She denied dysphagia, dyspnea, fever, night sweats, or weight loss. Her medical history was remarkable for multiple episodes of chylothorax in the past. The last one was seven years ago. Despite extensive workup, no causes for these chylothorax episodes could be identified. She has no history of hormone therapy. The surgical history was unremarkable. The patient denied tobacco, alcohol, and drug abuse. She was not taking any medication or oral contraceptives.

The inspection and palpation of the neck revealed a big cervical mass on the left side. The mass wasn’t red, warm, or painful to palpation, and the fiberoptic laryngoscopy was unremarkable.

**Figure 1:**
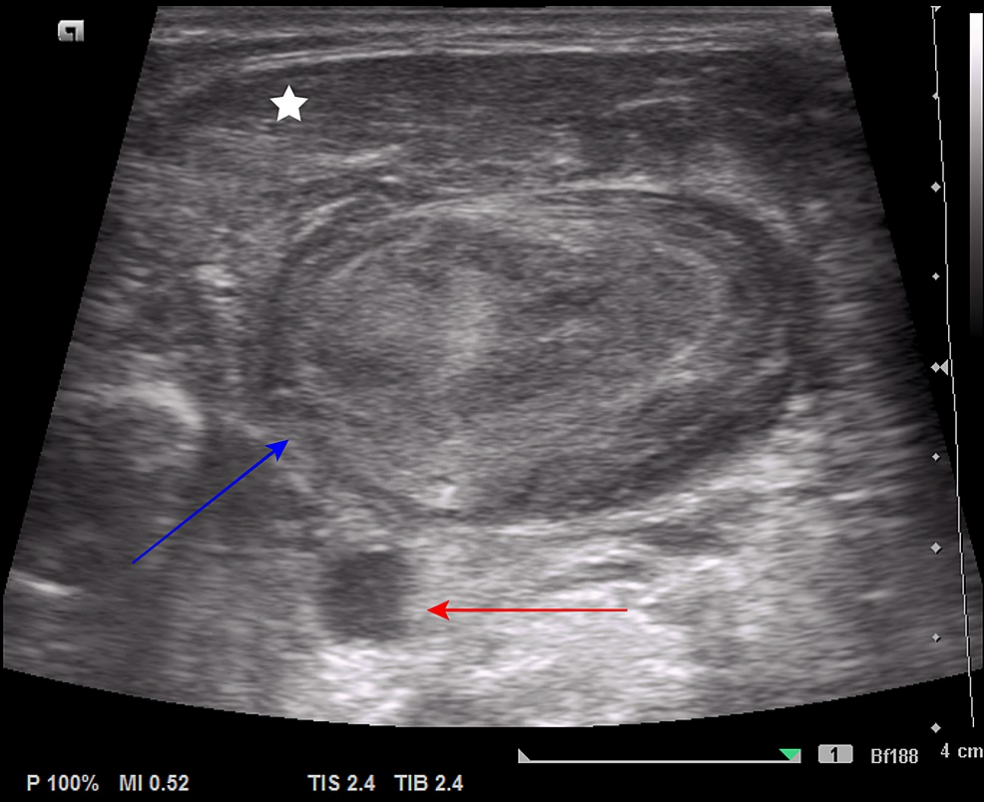
Ultrasound of the left side of the neck It shows the sternocleidomastoid muscle (star), the thrombotic left internal jugular vein (blue arrow), and the left common carotid artery (red arrow).

A cervical ultrasound revealed a huge iso-hypoechogenic mass underneath the whole course of the left sternocleidomastoid muscle from the skull base to region IV of the neck (Figure 1). Laboratory blood tests revealed leukocytosis (10.8 G/l) and elevated C-reactive protein (CRP) (35 mg/l).

**Figure 2:**
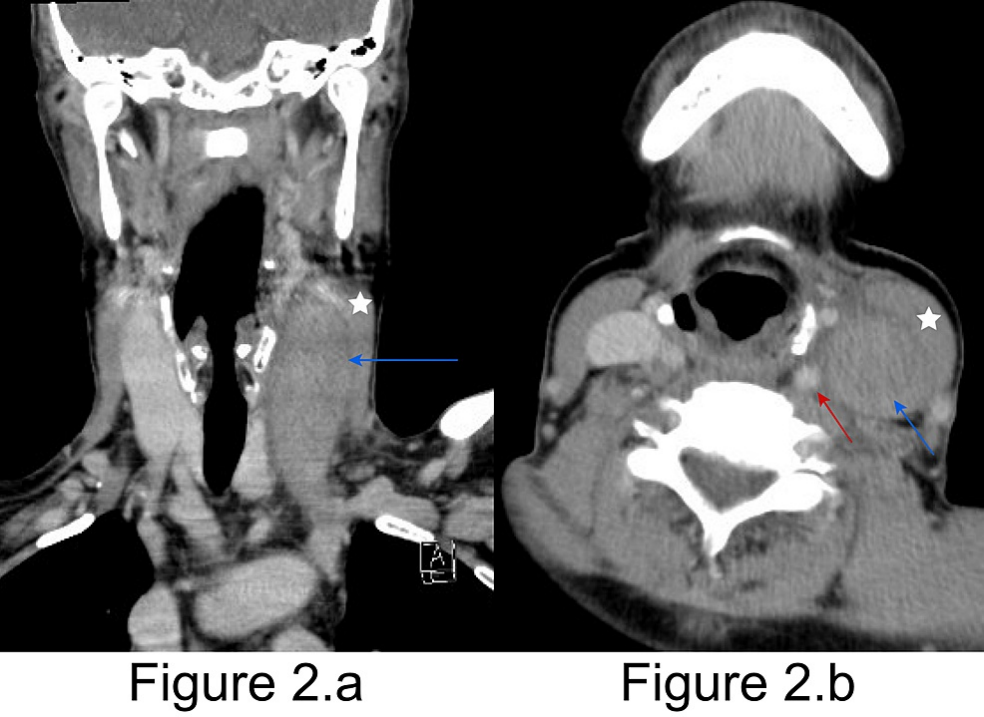
CT neck with contrast 2.a: Coronal view of the CT neck with contrast shows the sternocleidomastoid muscle (star) and the thrombotic left internal jugular vein (blue arrow). 2.b: Axial view of the CT neck with contrast shows the sternocleidomastoid muscle (star), the thrombotic left internal jugular vein (blue arrow), and the left common carotid artery (red arrow).

We suspected the obliteration of the internal jugular vein, as we could not identify its lumen with ultrasound. CT neck with contrast showed a big clot (2.7 x 2.2 x 11 cm) within the left internal jugular vein and ruled out abscess formation (Figure 2).

Vascular surgery and angiology consultations recommended the initiation of bridging anticoagulation with low molecular weight heparin (enoxaparin 40 mg/0.4 mL BID). We admitted the patient and started the recommended treatment. A duplex/Doppler ultrasound determined the limitation of the thrombosis to the left internal jugular vein, whereas the subclavian, anonymous, and external jugular vein were free of clots. However, the later imaging modality was not suitable for excluding intracranial thrombosis. Therefore, MRI phlebography was performed to exclude the cerebral venous sinus thrombosis.

An abdominal contrast-enhanced ultrasound (CEUS) excluded intrahepatic thrombosis or neoplasms. A cardiac consultation, including echocardiography, as well as a gynecologic consultation, including mammogram and breast ultrasound, were unremarkable. The ovarian hyperstimulation syndrome seems to be very unlikely in the absence of hormone therapy.

Extensive laboratory testing ruled out factor V mutations and vasculitis. Furthermore, the anticardiolipin antibodies and anti-beta 2 glycoprotein 1 antibodies were within normal ranges. A further molecular genetic analysis excluded mutations of the JAK2 (V617F) gene.

Eventually, the patient was diagnosed after counseling with internal medicine and vascular surgery for idiopathic IJVT. We discharged her with rivaroxaban (20 mg PO qDay) anticoagulation. The patient was recommended to continue anticoagulation for at least three months.

Two months' follow-up with duplex/Doppler ultrasound showed minimal regress of the thrombosis.

## Discussion

IJVT is potentially a life-threatening condition that is more common in women. Nonetheless, the rarity of this condition makes most clinicians unaware of its presentation. The scarcity of this pathology is probably responsible for the lack of epidemiological studies about it [2-4]. Provoked IJVT is more common and is associated with ENT procedures, head and neck cancer, pacemaker placement, and central catheter insertion [2,4]. The ovarian hyperstimulation syndrome seems to be a predisposing factor to unprovoked IJVT [3-4].

Most patients present with painful erythematous neck swelling and headache. However, the presenting symptoms could include also fever, facial swelling, papilledema, and signs of intracranial pressure elevations or neurological impairment [2,4].

The clinicians should be aware that the presenting symptoms could differ depending on the extent of the thrombosis involvement of the IJV, the presence of CVST, and the presence of bilateral IJV thrombosis [4].

In the clinical exam, the abrupt swelling of the neck and the palpation of a cord beneath the sternocleidomastoid muscle should raise suspicion for IJVT and mandate further imaging studies to confirm the diagnosis [2,4].

The cervical ultrasound is cost-effective and the quickest method to diagnose IJVT [2,4]. In our case, even though the cervical ultrasound results raised the suspicion for IJVT, we couldn’t confirm the diagnosis straight after the test, probably due to the lack of expertise in diagnosing such entities.

Therefore, we ordered a CT neck with contrast to exclude neoplasms or abscess formation - given the elevated infectious parameters - and to confirm the diagnosis. D-dimer should be done before proceeding to CT imaging whenever thrombosis is suspected, as it has a high negative predictive value. However, a negative D-dimer test cannot rule out thrombosis in patients with suggestive symptoms and predisposing conditions [5]. In our case, the need to exclude the abscess formation made the CT necessary anyway, so we did not order the D-dimer levels.

After the affirmation of IJVT and the initiation of treatment with anticoagulation, an assessment of the extent of thrombosis and the presence of further deep vein thrombosis (DVT) must be performed [2,4].

While the duplex/Doppler ultrasound is the best initial test to define the extent of the thrombosis and the presence of DVT, it still cannot identify intracranial thrombosis involvement [4,6]. We ruled out the intracranial venous thrombosis through MRI with venography. This imaging modality is recommended by the European Stroke Organization guidelines to diagnose intracranial venous thrombosis [5,7].

There isn’t a clear recommendation to screen patients with IJVT or CVST for thrombophilia or potential uncovered cancer [5]. Good clinical practice for unprovoked IJVT workup would exclude both of these predisposing conditions. In our case, we couldn’t identify any thrombophilia after an extensive laboratory workup.

To exclude any covered malignancy, the patient obtained gynecological consultation, fecal occult blood test, and CEUS. All of them were uneventful. Our patient was not taking human chorionic gonadotrophin so that we ruled out ovarian hyperstimulation syndrome. 

There are no specific guidelines to treat IJVT, however, the mainstay of the therapy is based on the guidelines for upper limb thrombosis therapy with anticoagulation for at least three months. The anticoagulation should be initiated with low molecular weight heparin for five to 10 days before switching to oral agents [2,6,8]. Continuation of the therapy beyond three months depends on the presence of thrombosis predisposing factors [6,8].

If the case were an IJVT combined with CVST, warfarin would have been the preferred anticoagulant (target INR 2.5-3) [4].

IJVT should be diagnosed and treated promptly, as it could lead to serious complications such as pulmonary embolism, stroke, DVT, and CVST [2-4]. Specific data about the prognosis of the IJVT are lacking, however, CVST shows a favorable prognosis if treated rapidly [4].

## Conclusions

The abrupt appearance of a cervical mass combined with palpation of a hard cord underneath the sternocleidomastoid muscle should raise a suspicion of IJVT. A cervical ultrasound by an experienced examiner can sufficiently confirm the diagnosis. However, a CT neck with contrast may be added to exclude predisposing factors, such as neoplasms or external compression of the IJV in the neck, and to ascertain the thrombosis. Anticoagulation must be initiated promptly to avoid severe complications. An extensive multidisciplinary workup is mandatory to define possible causes and further management.
